# Reply to Chi et al.: Defining *BRCA1P1* as a tumor-intrinsic immunoregulatory axis in breast cancer

**DOI:** 10.1073/pnas.2619005123

**Published:** 2026-07-13

**Authors:** Yoo Jane Han, Olufunmilayo I. Olopade

**Affiliations:** ^a^ Section of Hematology/Oncology, Department of Medicine, University of Chicago, Chicago, IL 60637; ^b^ Department of Human Genetics, University of Chicago, Chicago, IL 60637

While our recent work has demonstrated a critical role for *BRCA1P1* in regulating innate immune defense and antitumor responses in preclinical breast cancer models, including normal and malignant patient breast tissues, patient-derived tumor organoids, and humanized mouse models (1), we recognize that fully translating *BRCA1P1* biology into a broadly targetable immunotherapy axis will require further development of pseudogene-aware transcriptomic approaches (2). Chromosome 17q21 abnormalities, including loss of heterozygosity, deletions, and amplifications, are common in breast cancer (3). This region harbors the tumor suppressor gene *BRCA1*, as well as a partially duplicated pseudogene *BRCA1P1* (4). Germline pathogenic or likely pathogenic variants in the *BRCA1* gene represent the most predictive risk factors for early-onset breast cancer (5, 6), whereas *BRCA1P1* negatively regulates an antiviral defense mechanism in cancer cells (1, 7).

Given the importance of the 17q21 region in female cancers, the genomic location of *BRCA1P1* suggests that this locus may have clinical significance. Notably, *BRCA1P1* is a chimeric pseudogene with homology to both *BRCA1* and *RPLP1* and generates a substantial fraction of RNase R-resistant RNAs, which makes short-read RNA-seq technically challenging for transcriptomic analyses of this locus. Future work will benefit from targeted long-read sequencing (8), junction-specific assays, and systematic comparisons of RNase R-treated libraries to more precisely define *BRCA1P1* transcripts. In addition, investigating the structural topology of *BRCA1P1* using SHAPE-seq will help define its secondary structures and interaction interfaces (9, 10). These steps together represent important next phases to refine the actionable *BRCA1P1* species and to strengthen the translational framework for *BRCA1P1*-targeted immunotherapy.

Heterogeneity and complexity of normal and tumor tissues present an additional caveat for transcriptomic analyses, but this can be partly addressed by rigorous pathological review followed by cell type–resolved bioinformatic approaches. We agree that single-cell RNA profiling and spatial transcriptomics could be used to define the spatial localization of *BRCA1P1* transcripts. In our study, RNA in situ hybridization on patient breast tissue samples successfully identified the subcellular localization of *BRCA1P1* in breast epithelial cells. Future work incorporating high-resolution spatial mapping (11) of *BRCA1P1* transcripts together with key tumor microenvironment (TME) marker proteins should further delineate the spatial heterogeneity of *BRCA1P1* expression and clarify how *BRCA1P1*-expressing tumor cells interact with immune cells within the TME.

To minimize RNA-mediated activation of innate immunity, we additionally used tumor models in which *BRCA1P1* was inactivated by CRISPR-Cas9 genomic knockout, thereby avoiding exogenous RNA perturbations in both the pancancer cell and humanized mouse model studies. Going forward, translational efforts should include small-molecule screening to identify *BRCA1P1* pathway inhibitors, subsequent optimization of lead compounds, and development of efficient delivery strategies to breast tumors to enable preclinical studies of *BRCA1P1*-targeted therapies.

These experimental and translational refinements may outline a clearer path to validate *BRCA1P1* as a tumor cell–intrinsic immunoregulatory target and to realize its potential as a clinically actionable axis for enhancing antitumor immunity. We believe that this combined framework will not only clarify *BRCA1P1* biology but also help establish general principles for translating immunoregulatory pseudogenes into safe and effective treatments.

## Data Availability

There are no data underlying this work.
